# Differentiation of Rat bone marrow Mesenchymal stem cells into Adipocytes and Cardiomyocytes after treatment with platelet lysate

**Published:** 2016-01-01

**Authors:** Farshad Homayouni Moghadam, Tahereh Tayebi, Kazem Barzegar

**Affiliations:** 1 Neurobiomedical Research Center, School of Medicine, Shahid Sadoughi University of Medical Sciences, Yazd, Iran; 2 Department of Cellular Biotechnology, Cell Science Research Center, Royan Institute for Biotechnology, ACECR, Isfahan, Iran; 3 Yazd Cardiovascular Research Center, Shahid Sadoughi University of Medical Sciences, Yazd, Iran; 4 Department of Applied Cell Science, School of Advanced Technologies in Medicine, Shahid Beheshti University of Medical Sciences, Tehran, Iran; 5 Head of the English Language Department, School of Medicine, Shahid Sadoughi University of Medical Sciences, Yazd, Iran

**Keywords:** Cardiomyogenic differentiation, Platelet lysate, 5-azacytidine, Mesenchymal stem cells

## Abstract

**Background:** Mesenchymal stem cells (MSCs) are multipotential cells and their therapeutic potency is under intense investigation. Studying the effect of different induction factors on MSCs could increase our knowledge about the differentiation potency of these cells. One of the most important sources of these factors in mammalian body is platelet. Platelet lysate (PL) contains many growth factors and therefore, it can be used as a differentiation inducer. In the present study, the effect of PL on differentiation of rat bone marrow MSCs into cardiomyocytes was studied.

**Materials and Methods:** To study the differentiation-inducing effect of PL, MSCs were treated with 2.5, 5 and 10% PL. Early results of this study showed that PL in high concentrations (10%) induces adipogenic differentiation of MSCs. Therefore, to evaluate differentiation to cardiomyocytes, MSCs were cultured in media containing lower levels of PL (2.5% and 5%) and then cardiomyogenic differentiation was induced by treatment with 5-azacytidine. Differentiation of MSCs was evaluated using direct observation of beating cells, immunostaining and real-time PCR techniques.

**Results:** The results of qPCR showed that treatment with PL alone increased the expression of cardiac alpha actinin (CAA) being predictable by earlier observation of beating cells in PL-treated groups. The results of staining assays against cardiac alpha actinin also showed that there were stained cells in PL-treated groups.

**Conclusion:** The results of the present study showed that PL is a powerful induction factor for differentiation of MSCs into different cell lines such as cardiomyocytes and adipocytes.

## Introduction

 Using stem cells for regenerative medicine has become an interesting therapeutic purpose for treatment of various cureless diseases including cardiac infarctions.^   1 ^ Several types of stem cells have been identified and their efficacy, safety and mechanism of therapeutic effect are still under investigation.^   2 ^ Of these, mesenchymal stem cells (MSCs) have gained much more attention because of their accessibility and easy cultivation methods. These cells can be harvested from several tissues such as bone marrow, adipose tissue, umbilical cord and placenta.   ^3^^-^^5^ It has been reported by many studies that bone marrow MSCs are multipotent and they could generate osteoblasts, chondrocytes and adipocytes after treatment with defined factors.   ^6^^,^^7^ Also, it has been reported that mesenchymal stem cells from different sources have different differentiation tendencies.   ^8^^,^^9^ So far, several methods have been studied regarding the ability of MSCs to generate cardiomyocytes such as co-culture with cardiomyocytes,^   10 ^ treatment with induction factors   ^11^^-^^13^ and treatment with 5-azacytidine, i.e. a synthetic analog of cytosine that changes the expression pattern of a group of genes involved in differentiation probably by suppressing DNA methylation.   ^14^^,^^15^ Furthermore, investigations for finding better differentiation methods are still in progress.

Platelet lysate (PL) has multiple growth and differentiation factors such as platelet-derived growth factor (PDGF), fibroblast growth factor (FGF), insulin-like growth factor (IGF), transforming growth factor beta (TGFβ), platelet factor 4 (PF-4 ), platelet-derived epidermal growth factor (PDEGF), and platelet-derived angiogenesis growth factor (PDAGF).   ^16^^,^^17^ Some of these factors such as FGF are proliferative factors while others including PDGF, TGFβ and IGF-1 are differentiation induction factors.   ^12^^,^^13^ The study by Behfar et al. reported that treatment with different kinds of induction factors could direct MSCs to differentiate into cardiomyocytes. They used a cocktail of induction factors consisting of TGFβ1, BMP-4, Activin-A, retinoic acid, IGF-1, FGF-2, α-thrombin and IL-6.^        11 ^ Interestingly, PL contains most of these factors; so, this study was designed to evaluate the effect of PL on cardiomyogenic differentiation of MSCs.

## MATERIALS AND METHODS

**Isolation and Culture of MSCs**

 Bone marrow mesenchymal stem cells were isolated from adult male Wistar rats. Briefly, rats were deeply anesthetized by intraperitoneal (IP) injection of ketamine and xylazine cocktail (80 and 12 mg/kg, respectively, Sigma- K113) and sacrificed by cervical dislocation. Then, their femurs and tibiae bones were carefully removed and separated from the surrounding soft tissues aseptically. Next, the two ends of each bone were cut and bone marrow was extracted by flushing DMEM medium using 2ml syringe. Then, the bone marrow was transferred and cultured in 10 cm plates in DMEM (Gibco, 12800-017) containing 100 U/ml penicillin/streptomycin (PAA), 100 U/ml L-Glutamine (PAA) and 15% FBS (FBS gold, EU approved, PAA). The medium was changed after two days of seeding and non-adherent cells were removed. Proliferated MSCs were resuspended using 0.25% trypsin-EDTA and cultivated again in new culture dishes. The medium was changed every 3–4 days until proliferation of MSCs. The use of animals was done in accordance with the Guide for the Care and Use of Laboratory Animals published by the US National Institutes of Health and approved by the Animal Care and Use Committee at Shahid Sadoughi Yazd University of Medical Sciences.

**Platelet Lysate Preparation**

Platelet-rich plasma (PRP) was used for preparing platelet lysate.  ^16^^,^^18^ Following deep anesthesia and cervical dislocation as described above, blood samples of 10 male rats were collected via cardiac puncture and transferred into sterile tubes containing 3.8% sodium citrate. PRP was collected after centrifugation at 800 rpm for 15 min at 25°C. Approximately, 10 ml PRP was collected from 100 ml whole blood using this technique. Lysis of platelets was performed by three cycles of freezing and thawing. Then, contents of the tubes were transferred to centrifuge tubes and centrifuged at 5000 rpm for 20 min. The supernatant was filtered using 0.2 μm filter, 3U/ml heparin was added to prevent clot formation and 1U/ml of insulin was added to enrich the product, then it was stored at -20° C until use.

**Induction of Differentiation**

**Figure 1:**
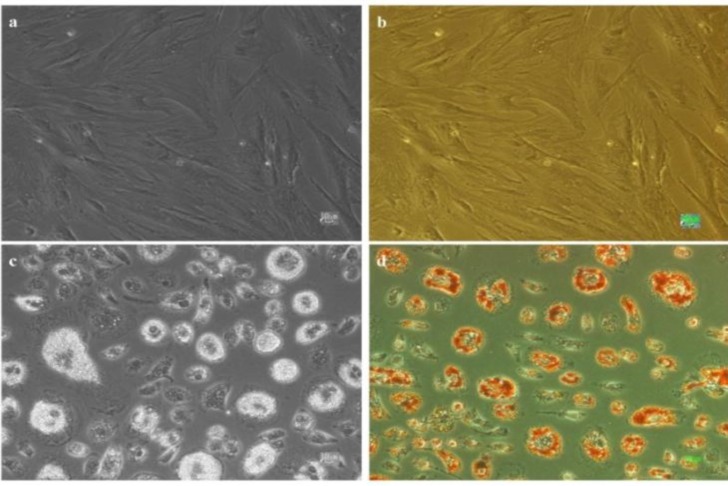
Generation of adipocytes from MSCs after one week of treatment with 10% platelet lysate, a) cells cultured in 5% FBS, b) cells cultured in 5% FBS after staining with oil red, c) cells cultured in 10% platelet lysate (PL) and d) cells cultured in 10% PL after staining with oil red. All scale bars represent 10 μm.

MSCs (P4) were used to evaluate their cardiomyogenic differentiation. For this purpose, approximately 15 × 10^4 ^cells were seeded in each well of 6-well culture dishes and treated with different medium supplements including 5% FBS as a control group and 2.5% PL and 5% PL in presence or absence of 5-azacytidine (Sigma, A2385) for cardiomyogenic induction. We used 2.5 and 5% PL concentrations because in our prior pilot studies, we found that PL with concentrations above 10% was adipogenic; so, lower concentrations of PL were used for cardiomyogenic induction (Figure 1). Six treatment groups were determined for this study: 1) 5% FBS, 2) 5% FBS + azacytidine, 3) 2.5% PL, 4) 2.5% PL + azacytidine, 5) 5% PL and 6) 5% PL + azacytidine. In azacytidine-treated groups, cells were treated with azacytidine (5 μmol for 24 hours) three days after seeding in PL or FBS-containing media. After azacytidine treatment, cells were cultured for 21 days (total culture period for all groups was 24 days). Culture media were replaced every 4-5 days. All experiments were performed three times.

**Cell Staining Protocols**

Cells were fixed in cold 4% paraformaldehyde for 10 minutes and then washed three times with washing buffer (0. 5% Tween 20 in PBS). Next, samples were incubated in 0.5% Triton X-100 (Sigma) in PBS for another 10 minutes. Subsequently, primary antibodies including anti-smooth muscle myosin (Rb pAb to smooth muscle myosin heavy chain 2, Abcam, ab 53219), anti alpha-actinin (Rb pAb to sarcomeric alpha actinin, Abcam, ab 90776) and anti skeletal muscle myosin (Sigma, M7523) diluted in dilution buffer (0.5% Tween 20, 1% BSA and 0.1% Sodium azide in PBS) were added to the samples with 1:100 concentration and incubated overnight at 4°C. Then, after three times washing, samples were incubated with secondary antibody (Goat pAb to Rb IgG, Abcam, ab 6718) at concentration of 1:100 for 2 hours. Finally, DAPI (Sigma, D9542) with the final concentration of 2 µg/ml in PBS was added to the samples to label the nuclei.

After incubation for 10 minutes, samples were washed three times. Images were captured from cells using an inverted fluorescence microscope equipped with digital camera (Olympus). For identification of lipid droplets inside the adipocytes, cells were stained by Oil Red O (Sigma, O0625). Briefly, cells were fixed by 4% paraformaldehyde, washed with ddH_2_O two times and then washed with 60% isopropanol. Afterwards, cells were incubated for 10 minutes in 0.002 g/ml Oil Red in 60% isopropanol and were finally washed with ddH_2_O for three times.

**Real-Time PCR**

Total RNA was extracted from cells using GF-1 

total RNA extraction kit according to the manufacturer’s instructions (Vivantis, Cat. No: GF-TR-025). Then, cDNA was synthesized from extracted RNA using MMLV reverse transcriptase and Oligo (dT) primer (Viva 2-steps RTPCR kit, Vivantis, Cat. No: RTPL 12).

Quantitative real time-PCR was performed by SYBR-Green-based method using Rotor-Gene Q (Qiagen) system. The expression rates of cardiac α-actinin (CAA), GATA4, Calponin, Smoothelin, MyoD, and Glyceraldehyde-3-phosphate dehydrogenase (GAPDH, housekeeping gene) mRNAs were evaluated. PCR was performed by 35 cycles of denaturating (94°C, 30s), annealing (52°C, 30s) and extension (72°C, 30s) temperatures. Real-time PCR SYBR Green Master Mix (Life Technologies, 4344463) was used for the amplification reactions. The sequences of primers are listed in Table 1. All procedures were repeated three times for each group.

**Statistical Analysis**

All results were expressed as mean ± standard error of mean (SEM). A comparative study of means was performed using one-way analysis of variance (one-way ANOVA) with post-hoc Tukey test using SPSS software (IBM Statistics, version 21). P-values less than 0.05 were considered as statistically significant.

**Table 1:** Primer sequences for real time PCR

Gene	Primer sequences	Pro. size	Gene ID
CAA	F: 5'- GGAGTCTTTCTGCCCCATACC-3' R: 5'- GCTGTACCAGGATGTGTGACG-3'	173bp	NM 019183.1
GATA4	F: 5'- CAGACCAAAGCGACAAGAATTG-3' R: 5'- TTGTTGCTCTGATGCTGGATTT-3'	129bp	NM 144730.1
Calponin	F: 5'-CAGGCAACTATCAGTCTACAGATG-3' R: 5'- GCGTGTCACAGTGTTCCAT-3'	121bp	NM 031747.1
Smoothelin	F: 5’- CGAGAAGCCGAGCAACAG-3’ R: 5'- CCGAAGCAGTGTGGTCAAC-3'	90bp	XM 008770259.1
MyoD	F: 5'- GCCATCCGCTACATTGAAGG-3' R: 5'- GCTGTAATCCATCATGCCATCA-3'	183bp	NM 176079.1
GAPDH	F: 5'- GTGGTCTCCTCTGACTTCAACAG-3' R: 5'- CTGTAGCCAAATTCGTTGTCATAC-3'	122bp	NM 176079.1

## Results

**Morphological Changes after Induction of Differentiation**

**Figure 2:**
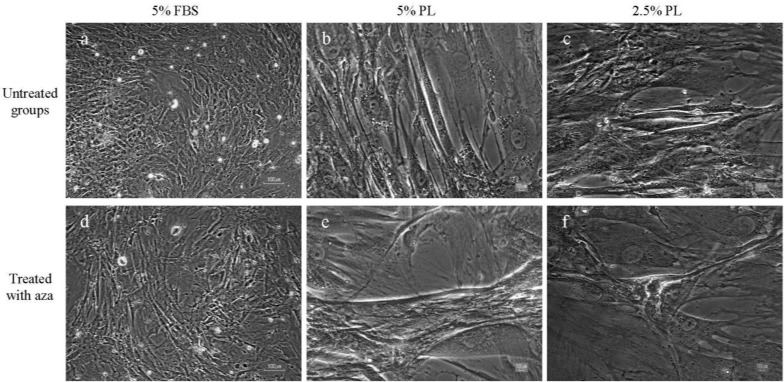
Morphology of mesenchymal stem cells after treatment with platelet lysate and 5-azacytidine (Aza) in different groups, MSCs cultured in **a)** FBS 5%, **d)** FBS 5% + Aza, **b)** PL 5%, **e)** PL 5% + Aza, c) PL 2.5% and f) PL 2.5% + Aza. Fibroblastic morphology of cultured cells in 5% FBS medium was maintained even after treatment with 5-azacytidine (a and d). MSCs cultured in PL containing media showed elongated spindle-like morphology one week after exposure to PL. These morphological changes were more apparent in 5% PL cultured cells (b and e) compared with 2.5% PL (c and f). Scale bars in **a** and **e** represent 100 μm and in other images 10 μm.

 Mesenchymal stem cells started to generate lipid droplets after one week culture in 10% PL and they was established by oil red staining (Figure 1). Morphological changes from fibroblastic to rod-like cells appeared gradually in groups cultured in 2.5 and 5% PL. Furthermore, cell migration and formation of integrated structures or cell aggregates were detectable in PL-treated groups. In contrast, cells that were cultured in the medium containing 5% FBS maintained their fibroblastic morphology (Figure 2).

Addition of 5-azacytidine dramatically changed the morphology of cells in the PL-treated groups. Apparently, in 5% PL + azacytidine-treated group, some cells started to form large aggregations during the first week of treatment, and some of them started to beat about two weeks after culture. In FBS and FBS + azacytidine-treated groups, there were not any detectable beating cells.

**Cell Staining Results**

**Figure 3:**
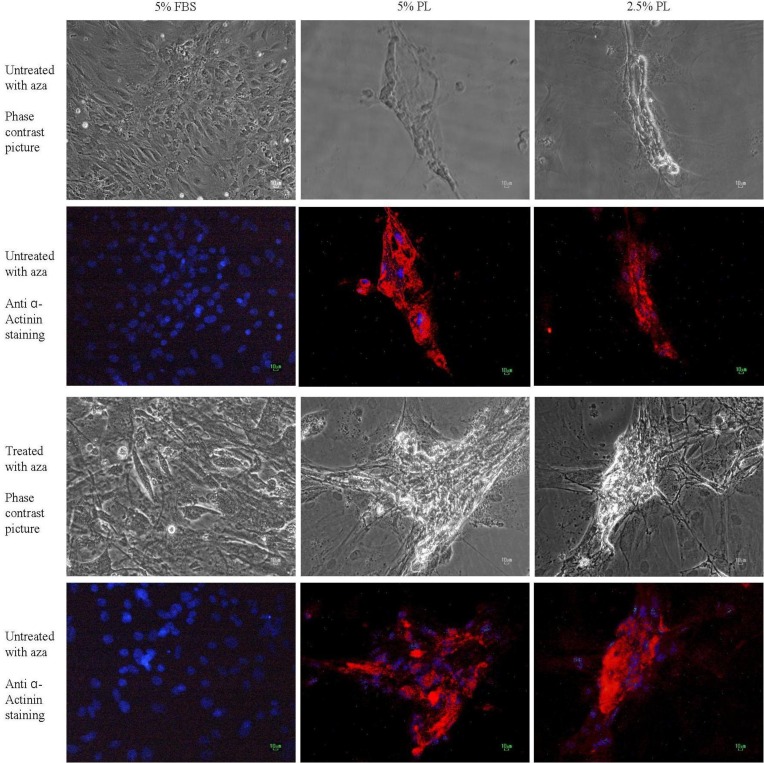
Immunocytochemical staining of cells against α-sarcomeric actinin after treatment with different concentrations of platelet lysate (2.5% and 5%) or FBS (5%) in presence or absence of 5-azacytidine (Aza). Red areas represent α-sarcomeric actin stained cells and blue areas (stained with DAPI) represent cells nuclei. Protein expression was detected in all PL groups and it was higher in groups treated with 5-azacytidine and PL .In the groups treated with FBS, there was not any staining against α-sarcomeric actinin. All scale bars represent 10 μm.

**Figure 4:**
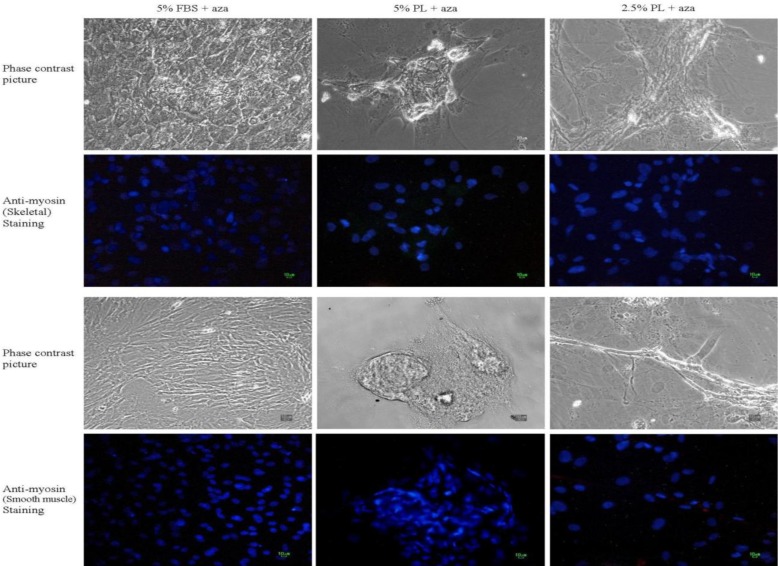
Immunostaining against skeletal myosin and smooth muscle myosin in MSCs after treatment with 5-azacytidine (Aza). There was no skeletal muscle or smooth muscle stained cell. All scale bars represent 10 μm.

Immunocytochemical staining against α-sarcomeric actinin, skeletal myosin and smooth muscle myosin was performed to evaluate the presence of muscle specific proteins in cultured cells. A remarkable positive staining was observed against α-sarcomeric actinin in the PL-treated groups (2.5 and 5%), while there were not any stained cells in 5% FBS-treated groups (Figure 3). No staining against skeletal muscle and smooth muscle myosin was observed in all of the treated and non-treated groups (Figure 4).

**Expression of Muscle Specific mRNAs**

**Figure 5:**
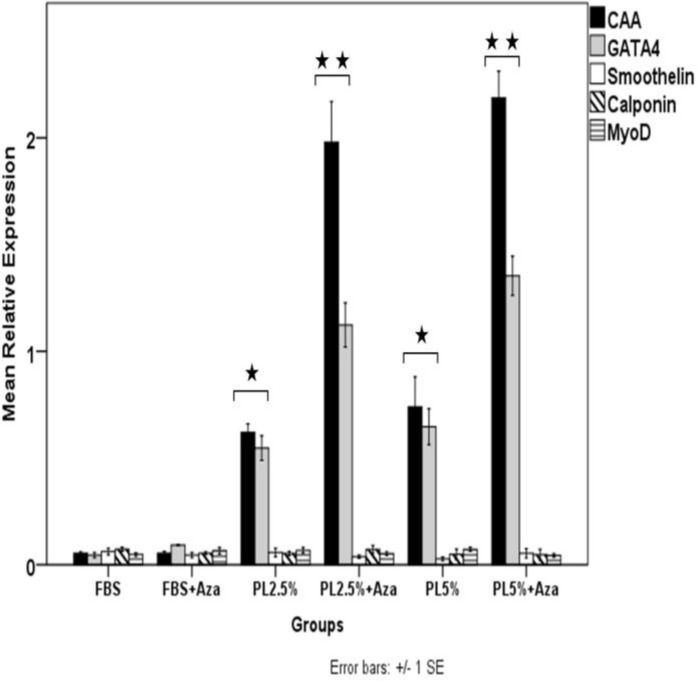
Relative expression of muscle specific markers. In the PL and PL + Aza (azacytidine) treated groups (both 2.5 and 5%), the relative expression of CAA and GATA4 mRNAs were remarkably higher than those in FBS and FBS + Aza-treated groups. Treatment with azacytidine significantly amplified the effect of PL on expression of CAA and GATA4 mRNAs in PL + Aza-treated groups compared with PL treated groups (P< 0.001). The expression levels of MyoD, smoothelin and calponin mRNAs were not significantly different among groups. There was no significant difference between PL 2.5% + Aza and PL 5% + Aza -treated groups. The expression level of each target gene was determined relative to the expression rate of the house keeping gene (GAPDH) in each group. Data were represented as Mean ± SEM. (*: P< 0.03; compared with FBS and FBS + Aza groups, **: P< 0.001 compared with FBS, FBS + Aza, PL 2.5% and PL5% groups).

As shown in Figure 5, there were remarkable changes in the expression pattern of cardiac specific markers in PL and PL + azacytidine-treated groups. Statistical tests comparing PL-treated groups (2.5 and 5%) with FBS-treated groups (FBS and FBS + azacytidine) confirmed that treatment with PL had significantly increased the expression of CAA and GATA4 mRNAs (P< 0.03). It was also determined that addition of azacytidine to the PL-treaterd groups (PL 2.5% + azacytidine and PL5% + azacytidine) had intensified this increase in the expression of cardiac specific markers (P< 0.001, comparing PL + azacytidine-treated groups with others). There was no significant difference in the expression pattern of smoothelin, calponin and MyoD mRNAs between different groups (Figure 5).

## Discussion

 Despite conflicting evidence regarding the ability of stem cells to generate cardiomyocytes, the cardiomyogenic differentiation ability of different types of stem cells such as MSCs has been reported by many studies.  ^13^^,^^19^^,^^20^ In line with them, the results of the present study demonstrated that MSCs could generate cardiac myocytes by exerting specific treatments on them. PL has different kinds of growth factors.  ^16^^,^^17^ It has been reported that these growth factors can direct differentiation of embryonic cells into multiple cell types including cardiomyocytes. For example, it has been reported that FGF and TGF-β could affect proliferation and development of cardiomyocytes;^   21^ moreover, the TGF-β and IGF-1 signaling pathways could be involved in the proliferation and regeneration of embryonic cardiomyocytes.^      22^ Furthermore, it was reported that treatment with a cocktail of multiple growth and induction factors in vitro can improve cardiomyogenic differentiation of stem cells after transplantation.^      11^ In one study, Behfar et al. reported that treating MSCs with multiple growth factors including FGFβ, BMP-4, IGF-1, alpha-thrombin and retinoic acid can promote human bone marrow MSCs to express cardiac specific markers such as CAA, troponin I, TBx5, MESP1 and MEF2c and differentiate them into cardiomyocytes.  ^11^^,^^12^ Other studies have also reported that A and B isoforms of platelet-derived growth factor (PDGF) could exert cardiomyogenic inductive effects on different kinds of stem cells.^      13^ Also, Vantler et al. reported that PDGFBB had anti-apoptotic and contraction-improving effects on engineered heart tissue.^   23^ The results of the present study also showed that PL can induce differentiation of MSCs into adipocytes in high concentration (10%), while it tends to differentiate them into myocardial cells in lower concentrations (2.5 and 5%). 

This adipogenic differentiation could be resulted from the presence of high levels of IGF-1 in 10% PL, as it has been reported that IGF1 is an adipogenic differentiation factor.^   24^ The effect of 5-azacytidine on induction of cardiomyogenic differentiation has been reported by many studies. Rangappa et al. evaluated the effect of different doses of 5-azacytidine by different exposure periods on adipose tissue-derived mesenchymal stem cells. They found almost 30% cardiomyogenic differentiation among cells.^   25^ Fukuda reported the same rate of cardiomyogenic differentiation after treatment with 5-azacytidine in mice MSCs.^      26^ However, Liu et al. reported that treatment with 5-azacytidine could not induce rat MSCs to form cardiomyocytes.^      27^ The findings of Liu et al.'s study were similar to the findings of the present study in the case of the cells cultured in the FBS-containing medium as they also used FBS + azacytidine for induction. So, according to the results of the present study, it is concluded that treatment with 5-azacytidine in the presence of FBS is not sufficient for cardiomyogenic differentiation of MSCs. The results of real-time PCR assay showed that CAA and GATA4 genes were highly expressed in PL-treated groups. CAA and GATA4 are known markers for cardiac myocytes.  ^19^^,^^28^^-^^30^ Evaluating the expression rate of smooth muscle and skeletal muscle specific genes (smoothelin, calponin and MyoD) showed that their expression was not considerable in treated cells. Smoothelin and calponin are known as smooth muscle-specific markers  ^31^^,^^32^ and MyoD is one of the most famous markers of skeletal muscle.  ^33^^,^^34^ The findings of immunostaining assays also approved the presence of alpha actinin protein in the PL-treated groups. Since there were high expression levels of CAA and GATA4 and very low levels of Calponin, smoothelin and MyoD in PL-treated groups and according to the results of staining assays, it can be concluded that the expressions of CAA and GATA4 were specific for cardiac muscle cells.

The presence of beating cells in PL-treated groups indicates that the results of real-time PCR and staining assays are reliable. The appearance of beating cells did not occur in a uniform pattern. As a result, their percentage was not measured in the present study. The innovative aspect of our findings was the generation of adipocytes and cardiomyocytes from MSCs by using PL. The results of this study also showed that PL in low concentrations is useful for the induction of cardiomyogenic differentiation and in higher concentrations it could generate adipocytes from bone marrow MSCs.

We propose further studies to evaluate the effect of PL in combination with other growth factors such as endothelial cell-derived growth factors to improve the method for production of cardiomyocytes from MSCs.

## CONCLUSION

 PL contains several types of growth factors and nutrients and can be used as a differentiation factor. Results of the present study showed that PL with defined concentrations can direct differentiation of mesenchymal stem cells into particular cell types such as cardiomyocytes and adipocytes.
